# Quality of life and activities of daily living one year after Coronary Artery Bypass Graft (CABG) surgery: a cross-sectional study

**DOI:** 10.1186/s13019-024-02848-y

**Published:** 2024-06-24

**Authors:** Khashayar Rezvani Emamzadehashemi, Atefeh Ghanbari Khanghah, Ali Azizi, Ezzat Paryad, Marzieh Jahani Sayad Noveiri

**Affiliations:** 1 Department of Neurosurgery, Faculty of Medicine, Imam Khomeini Hospital, Urmia University of Medical Sciences, Guilan University of Medical Sciences, Rasht, Iran; 2 Social Determinants of Health Research Center (SCHRC), School of Nursing and Midwifery, Guilan University of Medical Sciences, Rasht, Iran; 3 Nursing and Midwifery Faculty of Guilan University of Medical Sciences, Daneshjoo Ave, Rasht, Iran; 4 School of Nursing and Midwifery, Guilan University of Medical Sciences (GUMS), Rasht, Iran; 5 Cardiovascular Diseases Research Center, GI Cancer Screening and Prevention Research Center (GCSPRC), Department of Nursing (Medical-Surgical), Rasht, Iran; 6 Department of Medical Surgery, School of Nursing and Midwifery, Guilan University of Medical Sciences, Rasht, Iran

**Keywords:** Coronary Artery Bypass, Activities of daily living, Quality of Life

## Abstract

**Background:**

Daily activities have been recommended to minimize the long-term complications of coronary artery bypass as one of the strategies to return to the normal activity level, the effectiveness of which needs further investigation. This study aims to determine the quality of life and activities of daily living one year after CABG.

**Methods:**

This cross-sectional study was performed on 206 patients who had undergone CABG for more than one year in 2018 in the north of Iran. The research instrument was a questionnaire including five sections, Data were analyzed using descriptive statistics and Chi2, Mann–Whitney U, Kruskal–Wallis tests, and a Logistic regression model.

**Result:**

The mean score of quality of life was 31.7 ± 2.04 of 12 to 48 possible scores. About Activities of Daily Living results showed 99.5% and 84.7% of the samples needed help with many of these activities respectively. The mean score of quality of life was significantly different based on sex (*p* < 0.018) and instrumental activity of daily living (*p* < 0.0001). A logistic regression model was used to determine the factors related to quality of life. The final model showed cross-clamp duration (OR = 0.33,*p* = 0.014), length of stay(LOS)in the intensive care unit(OR = 0.42,*p* = 0.05), and instrumental activities of daily living (OR = 0.08,*p* = 0.001) predicted patients' quality of life one year after coronary artery bypass grafting.

**Conclusion:**

Although more than half of the samples had a good average quality of life score, due to the lack of definitive treatment for coronary artery disease, it is suggested to consider predictive variables to help plan to improve the quality of life of these patients.

## Highlights

• Daily activity is very important in patients undergoing heart bypass surgery.

• The quality of life in these patients has a significant relationship with their daily activity level.

## Plain language summary

The quality of life (QoL) of patients after CABG depends on many variables, one of the effective variables is the daily activity of these patients, which can significantly affect their quality of life, so it seems that by creating low-cost programs to improve the activity level of these people, it is possible to help increase their quality of life. The results of this research showed that about half of the research samples had an average quality of life and the majority of them still needed help to perform daily life activities. Maybe the design of special tools to determine the quality of life of these patients after coronary artery bypass surgery can show a clearer picture of the quality of life of these patients after surgery.

## Introduction

Coronary artery disease is one of the most common chronic diseases in the world which can be associated with mortality and 17.9 million people worldwide die each year from this disease [1]. In Iran, 97% of all patients have at least one cardiovascular risk factor including blood pressure, smoking, diabetes, high cholesterol, and low HDL levels. Treatment of the signs and symptoms of this disease includes medical treatments, and percutaneous and surgical interventions [2]. If the use of medical techniques cannot prevent the progression of the signs and symptoms, the use of Coronary Artery Bypass Graft surgery (CABG) is necessary to prevent the progression of the disease and improve the patient's quality of life [3]. Considering the extent of the surgery and the length of the patient's stay in the hospital after the surgery, doing the daily life activities of these patients after the surgery may be accompanied by difficulties. However in some centers, in order to minimize the complications after this surgery and Its effects on the daily life abilities of patients and their quality of life, rehabilitation programs start from the time of hospitalization and after surgery [4]. Numerous sources have emphasized the positive impact of activity on reducing postoperative complications, increasing life expectancy, and decreasing postoperative hospitalization after CABG [5–7]. In addition to reducing physical complications, physical activity can correct risk factors and improve the psychological and social condition of patients after surgery. [8].

Even though the patient's functional capacity is expected to improve rapidly with the improvement of the symptoms and signs of the disease following heart surgery, the results of some studies show that the functional capacity of the patients is not completely improved in the first months after the surgery [9]. Find in this case, we can refer to the findings of Benunotti et al. in their study, they found that the functional capacity of patients is still low three months and one year after coronary artery bypass surgery [10, 11]. This is despite the fact that some studies have reported that the ability of these patients to take care of themselves after surgery is favorable [11, 12].

According to various studies, different factors can affect the daily functioning of patients after CABG surgery. Recovery after coronary artery bypass surgery is a dynamic process and the impact of physical, mental-psychological and social factors on it includes a wide range of recovery to re-hospitalization and even death. The presence of mental-psychological disorders such as anxiety and depression in the stages before and after surgery will have a negative impact on the lives of patients within 6 months after surgery [13]. Age, sex, body mass index, level of education, living situation (alone, with spouse, with spouse and children), underlying diseases, cognitive status before and after surgery, anxiety and depression, patients' beliefs about their disease, existence of a support system, the type of surgery (with pump and without pump) and intraoperative risk factors (temperature during pump, aortic clamping time and duration of pump use) have been mentioned in different studies as factors that can affect functional status [10, 14–17].

Following this surgery and the complications that arise, these patients may experience a change in their ability to perform daily life care [10, 11, 18, 19]. Considering that the results of various studies have found different influencing factors on the patient's ability to perform daily life care, the patient's inability to perform self-care leads to the loss of a part of the workforce and the occurrence of economic losses for both the society and the patient. will lead [20, 21].

It seems that maintaining the daily self-care ability after heart surgery makes the patient return to society faster reduces the signs and symptoms of the disease, and also reduces the rate of re-hospitalization of these patients due to the problems they have. Therefore, the researcher decided to conduct research with the aim of determining the status of daily life activities and factors related to them in patients undergoing coronary artery transplant surgery referred to Dr. Heshmat's training and treatment center in Rasht City. The findings of this research can help nurses to identify the factors that can affect the patient's ability to provide basic self-care so that based on that, they can create a suitable care and education program for the patient and his family to continue care at home and after. In addition, due to the lack of a regular and structured rehabilitation system for these patients in many medical centers, the results of this research can help design an educational and care program for the patient and his family.

## Methods

### Setting and samples

This cross-sectional study was conducted on 206 patients who had undergone CABG for at least one year in the only government-specialized heart surgery center in one of the northern provinces of Iran in Rasht in 2018. To estimate the sample size to determine the relationship between daily life activities and related factors in patients undergoing coronary artery bypass surgery, we acted based on the results of the Duruturk study. According to the analysis of the results of this study, the correlation between the physical component of the occupational performance measurement tool and the movement component of Nottingham's daily life activities tool was -0.406 [22]. With 95% confidence and 90% test power, 155 people were selected according to the following sampling formula in a two-sided test. Due to data gathering by telephone call and the possibility of a 25% drop, the number of samples increased to 206. Sampling was performed by convenience sampling method.

$$Z1-\propto /2\to 1/96 , Z1-\beta \to 1/28$$

$${\text{N}}_{0}=\frac{{({\text{Z}}_{1-^{\propto }\!\left/ \!_{2}\right.}+{\text{Z}}_{1-\upbeta })}^{2}}{{(\frac{1}{2}\text{ Ln}\frac{1+\text{rxy}}{1-\text{rxy}})}^{2}} +3 \to {\text{N}}_{0}=\frac{10/4976}{0/184}+3=60/05$$

$${N}_{i}=\frac{{N}_{0}}{1-{r}_{i}^{2}} \to {N}_{i}=\frac{139/77}{0/906}=154/27$$

$${N}_{f}=\frac{1}{1-f}\times {N}_{i}\to {N}_{f}=\frac{1}{0/75}\times 154/27=205/69$$

All this information was collected from the medical documents registered in the archives of the educational-therapeutic center where the research was conducted, in such a way that after identifying each patient as a part of the research, the information was extracted from the patient archives. Inclusion criteria included at least one year has passed since the CABG, absence of any physical problems including motor, hearing, speech, and visual disorders based on their medical documents, failure to have another concomitant surgery with CABG, lack of use of drugs affecting the nerves due to mental disorders, all based on patient's medical records. The desire to not continue participating in the study and incomplete filling of the questionnaire were considered as exclusion criteria.

### Measurement

The research tool was a questionnaire consisting of these parts: The first part consisted of demographic characteristics. The second part of the questionnaire included clinical variables (including medications used based on their expression and medical records, the number of heart vessels involved, ejection fraction, and duration of connection to the cardiopulmonary bypass pump, duration of post-surgical intubation, central body temperature while receiving the cardiopulmonary bypass pump). After designing the first and second parts of the tool, including demographic characteristics and related clinical variables, the tool was tested by 10 members of the nursing academic staff with experience working in cardiac surgery and internal medicine departments and the nurses working in these departments, and the necessary modifications were made based on their opinions.

The third part was the six items Cognitive Impairment Test (6CIT). This test was developed in 1983and is useful for dementia screening. The answer to each question is based on a Likert scale, so that questions 1, 5, 6 have a score range of 0–4, questions 2, 4 have a score range of 0–3, question 3 has no score, and question 7 has a score range of 0–10. Therefore, the range of scores of this tool is in the range of 0–28, which is classified into three categories: 0–7 no cognitive impairment, 8–9 mild cognitive impairment, and 10–28 severe cognitive impairment [23]. The validity of this tool in Farsi was confirmed by ten professors specializing in the field of cognitive disorders, including psychiatrists, psychologists, and psychiatric nurses who are members of the academic faculty of the university. In order to determine the reliability of the method, re-testing was done after two weeks and the tool was asked over the phone from 15 patients who had been undergoing coronary transplant surgery for about a year with an interval of two weeks. The results confirmed the correlation of answers in two stages (*r* = 0.76).

The 4th part consisted of the Katz index tool instrumental daily activities of life questionnaire. Katz's questionnaire has 7 parts, each part has 3 answers, which are divided into independent, needing help, and dependent. Scoring is from 0 to 2, 2 points are assigned to the independent part, 1 point is needed for help, and 0 points are assigned to the dependent part. The overall score is 0 to 14 and is divided into three categories, 0 to 6 completely dependent, 7 to 10 needing help, and 11 to 14 completely independent.

The 5th part was the Instrumental Activities of Daily Life questionnaire which has 9 sections; each section has 3 answers, divided into independent, needing help, and dependent. Tool score from 0 to 18, divided into three categories, 0 to 8 completely dependent, 9 to 13 needing help, and 14 to 18 independent. These instruments were psychometrically translated into Farsi by Taheri Tanjani and Azad Bakht [24]. The findings of the psychometric study of Taheri and Azad Bakht showed that the tools of daily life activities and the daily activities of life tools are able to distinguish different age groups. The sensitivity and specificity for the questionnaire of daily life activities were 0.75 and 0.96, respectively, and for daily instrumental activities, 0.71 and 0.77 respectively. Cronbach's alpha and intraclass correlation were more than 0.75 for both tools. The presence of underlying diseases will be checked using Charlson's comorbidity index. This index has 19 questions that examine the presence of underlying diseases in the patient [25]. The last part was a short form12 (SF-12) health survey in adults. In this study, the psychometrically validated Persian form of this tool was used [26]. This questionnaire includes 12 questions that assess the patient's quality of life in terms of general understanding of their health (question 1), physical performance (questions 2, 3), physical health (questions 4, 5), physical problems (questions 6, 7), examines physical pain (question 8), social function (question 9), vitality and vital energy (question 10) and mental health (question 11, 12). The total score of the questionnaire is calculated and obtained from the sum of the scores related to 12 questions. In other words, the number in front of each answer is the score corresponding to that question. Also, questions 1, 8, 10, 11 are scored in reverse. The range of scores of this questionnaire varies from 12 to 48. According to the scores they get in the questionnaire, people are classified into three categories: poor (12–24), average (25–36), and good (37–48). Therefore, earning more points means a better quality of life.

### Data collection

Due to the lack of a system to follow up patients after heart surgery in the data collection center, the researchers used telephone calls to collect data on patients who underwent surgery for at least one year. For this reason, after receiving the approval of the Ethics Committee and obtaining permission to collect the data, they were contacted based on the numbers cited in the patient's file in the hospital archives. Data collection was done in the winter of 2018 and patients who underwent surgery during 2017 and had undergone surgery at least one year at the time of the study were included in the study. Data were collected by contacting 290 telephone numbers and finally collecting data on 206 individuals. All samples expressed their verbal consent to participate in the study. To collect data in this study, 290 patients whose telephone numbers were registered in the registry department of the educational and medical center of the research site and at least one year had passed since their surgery was called. Both the landline and mobile phone numbers of the patients were registered in the admission ward, and if the landline number was not answered, the mobile phone would be called.

### Data analysis

Data were analyzed using descriptive statistics, Kolmogorov–Smirnov test to determine the normal distribution of data and Chi2, Exact fisher, Mann–Whitney U, Kruskal–Wallis tests and logistic regression model by SPSS version 16.

### Ethical consideration

The study was approved by the Ethics Committee of Guilan University of Medical Sciences (IR.GUMS.REC.2018.108). After obtaining the necessary permissions the researchers stated gathering data. Due to the lack of face-to-face access to the study participants, oral consent was obtained from all of them to participate in this study.

## Results

The results showed that the majority of the 206 samples were less than 60 years of age and their mean age was 59.84 ± 8.51. The majority were men (69.9%), did not smoke (66%), were married (97.6%) and were literate (45.1%). Most had their business (63.5%), living with their spouses (50.4%) and were overweight (44.6%). The disease and surgical characteristics of the samples are presented in Table 1.

**Table 1:** Distribution of demographic, disease and surgical characteristics of samples (*n* = 206)

Variables		no(%)	Mean ± SD
**Ejection fraction (%)**	≤ 50	156(75.7)	44.41 ± 10.05
	> 51	50(24.3)	
Aortic cross clamp time(min)	≤ 36	118(57.3)	35.74 ± 11.67
	> 37	88(42.7)	
Cardiopulmonary bypass time(min)	≤ 60	122(59.2)	58.72 ± 16.63
	> 61	84(40.8)	
Length of stay in ICU(h)	≤ 59	132(64.1)	58.46 ± 18.71
	> 60	74(35.9)	
Intubation time after CABG	≤ 595.70	114( 55.3)	595.70 ± 174.3
	> 595.70	92(44.7)	
Job	Unemployed	13(6.3)	
	Employee	7(3.5)	
	Retired	33(16)	
	House keeping	64(31.1)	
	Self-employed	89(43.2)	
Education	Illiterate	77(37.4)	
	Reading and writing	93(45.1)	
	High school	28(13.6)	
	university	8(3.9)	
Living status	Alone	18(8.7)	
	With a spouse	103(50)	
	With children	13(6.3)	
	With spouse and children	72(35)	
Body Mass Index	< 25	70(34)	
	25.1–30	92(44.2)	
	> 30.1	45(21.8)	
Number of grafts	1	1(0.5)	
	2	26(12.6)	
	3	101(49.0)	
	4	66(32.0)	
	5	12(5.8)	

The results on the cognitive status of the samples showed that the majority of the samples (61.6%) did not have cognitive impairment, but 38.4% of them had some degree of cognitive impairment. Results in samples without cognitive impairment showed that 99.2% of them needed help with daily living activities.90.6% of samples without cognitive impairment needed help with instrumental activities of daily living. Results showed cognitive status was significant based on sex (*p* < 0.0001), smoking history (*p* < 0.0004), marital status (*p* < 0.03),job (*p* < 0.0001), living status (*p* < 0.017) and education status (*p* < 0.0001)(Table 2).

**Table 2:** Distribution of cognitive status based on demographic, disease and surgical characteristics (*n* = 206)

Cognitive status		did not have cognitive impairment	Moderate cognitive impairment	Severe cognitive impairment	*p*
Sex	Male	102(49.5)	14(6.8)	28(13.6)	0.0001*
	Female	25(12.1)	9(4.4)	28(13.6)	
Smoking history	Yes	54(26.2)	4(1.19)	12(5.8)	0.004*
	No	73(35.4)	19(9.2)	44(21.4)	
Marital status	Single	5(2.4)	0(0)	0(0)	0.03**
	Married	12(59.2)	23(11.2)	56(27.2)	
Job	Unemployed	9(4.4)	0(0)	4(1.9)	0.0001**
	Employee	1(0.5)	1(0.5)	0(0)	
	worker	3(1.5)	1(0.5)	1(0.5)	
	Retired	25(12.1)	2(1)	6(2.9)	
	housewife	22(10.7)	12(5.8)	30(14.6)	
	Self-employment employment	67(32.5)	7(3.4)	15(7.3)	
Living status	Alone	10(4.9)	1(0.5)	7(3.4)	0.017**
	With wife	61(29.6)	13(6.3)	29(14.1)	
	With children	3(1.5)	3(1.5)	7(3.4)	
	With spouse and children	53(25.7)	6(2.9)	13(6.3)	
Education status	Illiterate	26(12.6)	12(5.8)	39(18.9)	0.0001**
	Reading writing	69(33.5)	11(5.3)	13(6.3)	
	High school diploma	24(11.7)	0(0)	4(1.9)	
	University	8(3.9)	0(0)	0(0)	
Ejection friction	$<50$	94(45.6)	16(7.8)	46(22.3)	0.3 *
	$\ge 51$	33(16)	7(3.4)	10(4.9)	
age	$<60$	80(38.8)	13(6.3)	21(10.2)	0.006*
	$\ge 61$	47(22.8)	10(4.9)	35(17)	
Body mass index	Healthy weight range	45(24.1)	5(2.7)	20(10.7)	0.5*
	Overweight	43(23.0)	12(6.4)	20(10.7)	
	Obesity	27(14.4)	4(2.1)	42(22.5)	
Activity of daily living	Help need	126(61.2)	23(11.2)	56(27.2)	0.9**
	Independent	9(0.5)	0(0)	0(0)	
Instrumental activity of daily living	Help need	115(56.7)	17(8.4)	40(19.7)	0.012*
	Independent	12(5.9)	6(3)	13(15.3)	
Graft number	1	1(0.5)	0(0)	0(0)	0.6**
	2	14(6.8)	1(0.5)	11(5.3)	
	3	61(29.6)	14(6.8)	26(12.6)	
	4	44(21.4)	7(3.4)	15(7.3)	
	5	7(3.4)	1(0.5)	4 (1.9)	
Aortic cross clamp time(min)	≤ 36	76(36.9)	15(7.3)	34(16.5)	0.8*
	> 37	51(24.8)	8(3.9)	22(10.7)	
Cardiopulmonary bypass time(min)	≤ 60	74(35.9)	13(6.3)	35(17)	0.8*
	> 61	53(25.7)	10(4.9)	21(10.2)	
Length of stay in ICU		85(41.3)	12(5.8)	35(17)	0.38*
		42(20.4)	11(5.3)	21(10.2)	
Intubation length(m)	≤ 595.70	36(17.6)	5(2.5)	13(6.4)	0.67*
	> 595.70	91(44.1)	19(8.8)	42(20.6)	

^*^Chi 2

^**^Fisher exact test

The results about Activities of Daily Living (ADL) and Instrumental Activities of Daily Living (IADL) showed 99.5% and 84.7% of the samples needed help with many of these activities respectively. Of course, none of the samples were dependent on other people for these activities. Based on the results Instrumental activity of daily living was significant based on sex (*p* < 0.0001), job (*p* < 0.0001) and living status (*p* < 0.031) (Table 3).

**Table 3:** Distribution of Instrumental Activity of daily Living based on demographic, disease and surgical characteristics (*n* = 206)

Instrumental Activity of daily Living		Need to help No, (%)	Independent N0.(%)	*p*
Sex	Female	41(19.7)	19( 9.4)	0.0001*
	Male	134(65)	12(5.9)	
Smoking history	Yes	63( 35.5)	9(29)	0.51**
	No	112(64.5)	22(71)	
Marital status	Single	6(2.9)	0 ( 0)	0.99**
	Married	169(97.1)	31(100)	
Job	Unemployed	9( 5.2)	4(1.9)	0.1
	Employee	1( 0.6)	1(3.2)	
	worker	5(2.9)	0 ( 0)	
	Retired	32 ( 18.1)	2( 6.5)	
	housewife	43( 24.4)	19( 61.3)	
	Self-employment	84(48.8)	6 (16.1)	
Living status	Alone	16(9.3)	2(6.5)	0.031*
	With spouse	88 (50.6)	16 (51.6)	
	With children	7( 3.5)	5 ( 16.1)	
	With spouse and children	65 ( 36.6)	8 ( 25. 8)	
Education status	Illiterate	59 ( 33.7)	16 (51.6)	0.24 *
	Reading writing	82 ( 47.1)	12 ( 38. 7)	
	High school diploma	26 ( 15. 1)	2 ( 6.5)	
	University	7 ( 4.1)	1 ( 3.2)	
Ejection friction	< 50	34 (77.3)	22 ( 67.7)	0.1**
	50	40(22.7)	10 ( 32.3)	
Age	< 60	99(48.8)	14 (6.9)	0.1**
	60	39(18.9)	17 (8.4)	
Body Mass Index	Healthy weight range	56 (36.9)	11(39.3)	0.9*
	Overweight	81 (40.8)	11(39.3)	
	Obesity	39 ( 22.3)	6 ( 21.4)	
Graft number	1	0 ( 0)	1 ( 0.5)	0.2**
	2	21 ( 10. 3)	6 ( 2.5)	
	3	87 ( 42.4)	12 ( 5.9)	
	4	54 ( 26.6)	12 ( 5. 9)	
	5	12 ( 5. 9)	1 ( 0.5)	
Activity of daily living	Help need	174 ( 84.7)	30 ( 14.8)	0.15*
	Independent	1(0.5)	1 ( 0.5)	
Aortic cross clamp time(min)	≤ 36	104 ( 50.2)	21 ( 10.3)	0.42*
	> 37	71 ( 34.5)	10 ( 4. 9)	
Intubation length(m)	≤ 595.70	49 ( 23.4)	6 ( 3)	0.38*
	> 595.70	126 ( 61.2)	25 ( 12.4)	
Cardiopulmonary bypass time(min)	≤ 60	104 ( 50.2)	18 ( 8.9)	0.9*
	> 61	71 ( 34.5)	13 ( 6.4)	
Length of stay in ICU(h)	≤ 59	116 ( 56. 2)	18 ( 8. 9)	0.24*
	> 60	59 ( 28. 6)	13 ( 6.4)	

^*^Chi 2

^**^Fisher exact Test

The mean score of quality of life was 31.7 ± 2.04 of 12 to 48 possible scores and the majority of samples (61.7%) scored higher than mean score. About Activities of Daily Living (ADL) and Instrumental Activities of Daily Living (IADL) results showed 99.5% and 84.7% of the samples needed help with many of these activities respectively. Of course, none of the samples were dependent on other people for these activities( Table 4).The results showed the mean score of quality of life was significant different based on sex ( *p* < 0.018) and instrumental activity of daily living (*p* < 0.0001). The mean score of the quality of life according to the marital status had a borderline significant difference (*p* < 0.059).

**Table 4:** Distribution of quality-of-life mean score based on demographic, disease and surgical characteristics (*n* = 206)

Quality of life		< Mean score	≥ Mean score	*p*
Sex	Female	33( 16)	29( 14.1)	0.018*
	Male	46( 22.3)	98( 48.6)	
Smoking history	Yes	27( 13.1)	43( 20.9)	0.35*
	No	52( 25.2)	84( 40.8)	
Marital status	Single	4( 1.9)	1( 0.5)	0.059*
	Married	75( 36.4)	126( 61.2)	
Job	Unemployed	9( 4.4)	4(1.9)	**0.41
	Employee	1( 0.5)	1(0.5)	
	worker	2 ( 1)	3( 1.5)	
	Retired	12(5.8)	21( 10.2)	
	housewife	33( 16)	31( 15)	
	Self-employment employment	22(10.7)	67(32.5)	
Living status	Alone	10(4.9)	8(3.9)	0.64**
	With wife	35 (17)	68(33)	
	With children	5( 2.4)	8(3.9)	
	With spouse and children	29 (14.1)	43 ( 20.9)	
Education status	Illiterate	34 ( 16.5)	43 (20.9)	0.75**
	Reading writing	30 (14.6)	63 (30.6)	
	High school diploma	11 (5.3)	17 (8.3)	
	Unemployed	4 ( 1.9)	4 (1.9)	
Intubation length(m)	≤ 595	19 (9.3)	15 (17.2)	
	> 596	60 (29.4)	90 (44.1)	
Ejection friction	< 50	58(282)	98 (47.6)	0.28 *
	51	21(10.2)	29 (14.1)	
age	≤ 60	40(19.4)	74 (35.9)	0.52*
	61	39(18.9)	53 (25.7)	
Body Mass Index	Healthy weight range	22 (10.8)	45(22.2)	0.46*
	Overweight	35 (17.2)	57 (28.1)	
	Obesity	21 ( 10.3)	23(11.3)	
Activity of daily living	Help needed	53(26.1)	23(11.2)	0.0001*
	Independent	23(11.3)	8 (3.9)	
Instrumental activity of daily living	Help needed	78(37.9)	127(61.7)	0.62*
	Independent	1(0.5)	0 (0)	
Graft number	1	0 ( 0)	1(0.5)	0.14**
	2	16(7.8)	10 (4.1)	
	3	36 (17.5)	65 (31.6)	
	4	22 (10.7)	44 (21.4)	
	5	5 (2.4)	7 (3.4)	
Aortic cross clamp time(min)	≤ 36	44(21.4)	81(39.3)	0.79*
	> 37	35 (17)	46(22.3)	
Cardiopulmonary bypass time(min)	≤ 60	42(20.4)	80(38.8)	0.34*
	> 61	37(18)	47(22.8)	
Length of stay in ICU(h)	≤ 59	44(21.4)	88(42.7)	0.38*
	> 60	35(17)	39(18.9)	

^*^Mann Whiteney U test

^**^Kruskal Wallis test

Logistic regression model was used to determine the factors related to quality of life. At this step, all variables that had p value less than 0.25 in univariate study were entered into the model. The model was run in 8 step and the final model showed cross clamp duration( OR = 0.33, *p* = 0.014), length of stay(LOS) in the intensive care unit (OR = 0.42, *p* = 0.05), and instrumental activities of daily living (OR = 0.08, *p* = 0.001) predicted patients' quality of life one year after coronary artery bypass grafting(Table 5).

**Table 5:** Variables related to quality of life based on logistic regression

Variable	B	S.E	Wald	df	Sig	Exp(B)	CI95% Lower–Upper
Aortic cross clamp time	-1.107	0.45	5.99	1	0.014	0.33	0.13–0.8
Length of stay in ICU	-0.86	0.46	3.69	1	0.054	0.42	0.17–1.01
Instrumental activities of daily living	-2.43	0.76	10.16	1	0.001	0.08	0.02–0.39
Marital status	2.21	1.20	3.34	1	0.06	9.13	0.85–97.71
Constant	4.25	3.008	1.99	1	0.15	70.14	

## Discussion

The aim of this study was to determine the status of quality of life, daily life activities, and factors related to patients undergoing coronary artery transplant surgery one year after surgery. The results showed that the majority of samples were in middle age and the majority of them had their own private business. In Iran, private business means that everyone earns money from their private business and does not have continuous government income. Therefore, it is necessary to be present at work at most hours. However, the results of this study have shown that the majority of the units surveyed needed help to maintain their ADL and IADL but some of them have not been completely dependent on others for their activities. It is important to note that the majority of the samples in this study did not have cognitive impairment and even the majority of people without cognitive impairment needed help in carrying out their daily activities. However, some researchers have suggested that the need to help with daily living after heart surgery is more common in patients with delirium after open heart surgery. In this regard the study by Guenther et al. showed that patients with delirium after open-heart surgery needed help with their daily activities six months after surgery [27]. The high need for these people to find help to perform daily activities in this study may be due to their high age and occupational status. The results showed that the majority of patients were in middle age and the majority of them had their own private business. In Iran, private business means that everyone earns money from their private business and does not have continuous government income. Therefore, it is necessary to be present at work at most hours. The findings of Khodadadi et al.'s study on cognitive status showed that the average cognitive status of elderly people who were unable to perform daily life activities was significantly higher than that of people who were unable to perform daily life activities. Also, the results indicated that the chance of cognitive impairment was higher in the elderly with the inability to perform daily life activities [28].

In the present study, all employed people had returned to their former jobs and in many cases were still dependent on the help of others to carry out their daily activities. The results of a study by Elzbieciale et al. on the ability of postoperative cardiac surgery in elderly patients showed that most of these patients were dependent on others for ADL and IADL [29]. The results of another study showed perfusion time, by cardiopulmonary bypass pump, was associated with poor IADL [30]. However, the results of a study by Smith et al., which examined patients one year after coronary artery bypass graft surgery, showed that most were independent or had difficulty but did not need help in ADLs and IADLs [31]. Many similar studies have been performed on very elderly patients. For example, a study of the quality of life of patients over the age of 85 after several years of coronary artery bypass grafting showed that most of these patients were not active in the community and were mostly at home [32], of course, these conditions can be affected by the age of the research samples. The results of a similar study that looked at patients' performance one year after heart surgery showed that the ability to function in the elderly was much lower than in younger patients. However, it should be noted that the mean age of the patients was 72 years in that study. Their mean age was higher than the mean age of the research units in this study. The authors of that study emphasized the need for daily activities after cardiac surgery [33]. The results of the study by Sumi et al. also showed that even up to 7 years after cardiac surgery, their samples need help to perform their activities of daily living [34]. The results of Bak and Marcize's study also confirmed the low quality of life in patients aged 60 and over, one year after coronary artery bypass graft surgery [35].

However, the results of a study on patients after CABG show that physical activity after surgery can increase muscle strength, which will certainly be associated with an increase in the patient's abilities after surgery [36]. But, the results of a study on determining the difference in quality of life scores before CABG, one month after that, and one year after that did not show a significant difference in the quality of life of patients who underwent regular physical activity programs after CABG [37].

The results of this study showed that despite the need to receive help for daily life activities, most of the samples evaluated their quality of life as average. It should be noted that the quality of life has a unique meaning for each person. Perhaps the reduction of the symptoms and signs of coronary artery disease, which caused many limitations in the patient's life, has been able to affect the way these patients look at their quality of life one year after surgery. Returning to work, the sense of independence due to earning income, even despite feeling the need to receive help for daily life activities, may have influenced the perception of these patients about the quality of life.

In the present study, even though the mean age of the samples indicated that most of them were in middle age, they still needed help to perform ADL and IADL. However, none of the samples in the present study received any program for daily activity and its follow-up. It is possible that daily activity programs could increase the empowerment to do ADL and IADL. Due to the lack of use of follow-up programs after discharge for patients after CABG, there is no accurate picture of their condition in our country. These patients only go to the cardiac surgery center if they have problems with surgery. It should be noted that many of these patients are in the early years of middle age and are a useful workforce in society. Lack of follow-up plans after surgery may cause irreparable damage. The results of the present study on the factors related to a patient's quality of life after one year of CABG showed that the duration of clamping aorta and greater ability to perform IADL predicted the quality of life. Due to the fact that the number of grafted arteries can affect the duration of using the cardiopulmonary bypass pump and the length of the aortic clamp, perhaps the long duration of the aortic clamp means more vascular transplants. Increased coronary artery bypass grafting may increase the patient's ability to perform life activities and improve their quality of life by increasing cardiac blood flow. However, no study was found on the long-term consequences of the length of the aortic clamp in CABG.

Regarding the relationship between increasing ability to perform IADL and quality of life one year after CABG, attention to instrumental expressions shows that most of these activities are consistent with the activities that the patient performs after returning to work. When patients see in their selves the ability to do the things that are necessary for them to do the job independently, it will probably affect their quality of life as well. The results of Bak's study showed that one year after surgery, the ability of men to perform IADL increased before they underwent CABG, and the relationship between IADL performance and adequate energy in women one year after CABG was significant [35].

## Implication and limitations

The use of telephone calls for data collection is one of the limitations of this research, but it should be noted that due to the lack of access to the research samples and the geographical dispersion of their residences, it was not possible to use the face-to-face method for data collection and Its application in nursing is to pay attention to creating cheap rehabilitation conditions for heart patients.

## Conclusions

The results of this research show that a large number of research samples feel the need to receive help to perform some of their daily life activities, even one year after coronary artery bypass surgery. This is despite the fact that the majority of these people have returned to their former jobs and the reason for returning in most cases was the need to earn income. These findings show the need to pay more attention to rehabilitation programs in these patients. Designing rehabilitation programs for patients after coronary artery bypass surgery can help to reduce patients' dependencies. The design of rehabilitation programs can play a special role in the career and professional success of these patients who have to return to their previous jobs due to their age range. Unfortunately, despite the confirmation of the effect of rehabilitation programs on improving self-care in these patients and reducing their dependence on caregivers, there is still no documented program for the follow-up of these patients and the design of a rehabilitation program by managers providing treatment and care services. It seems that designing cost-effective follow-up and rehabilitation programs and monitoring their optimal implementation by managers can prevent the wastage of these social capitals, i.e. the effective workforce. Therefore, it is suggested that in future studies, economic solutions should be investigated in the design of rehabilitation programs for these patients.

## Data Availability

No datasets were generated or analysed during the current study.
